# Bioremediation potential of low-brominated polybrominated diphenyl by the phyllospheric *Wickerhamomyces anomalus*

**DOI:** 10.3389/fpls.2025.1606962

**Published:** 2025-07-11

**Authors:** Man Cai, Zheng Xing, Xiaolei Yuan, Zhangyu Song, Xiaojing Wang, Kejiu Du

**Affiliations:** ^1^ College of Forestry, Hebei Agricultural University, Baoding, China; ^2^ Hebei Key Laboratory for Tree Genetic Resources and Forest Protection, Baoding, China

**Keywords:** PBDEs, phyllosphere microbiota, ABC transporter, vacuolar sequestration, bioremediation

## Abstract

Phyllospheric microorganisms play a significant role in environmental bioremediation. However, there have been limited studies to assess the detoxification mechanisms of phyllospheric *Wickerhamomyces anomalus* in persistent organic pollutants (POPs), especially polybrominated diphenyl ethers (PBDEs). In this study, we performed RNA sequencing (RNA-seq) to identify the detoxification mechanism of 4-monobrominated diphenyl ether (BDE-3) and to identify abundant genes, differentially expressed genes, and promising candidates in phyllospheric yeast. The transcriptome analysis revealed that the potential detoxification genes were classified into four clusters: cell-wall binding, complexation, vacuolar sequestration, and efflux. The aim of this research was to study the functions of overexpressing heterologous yeast genes in plants. We identified possible candidate genes that may maintain high expression during vacuolar sequestration. The WICANDRAFT_64792 gene is a member of the ABC transporter family. The overexpressing *WICANDRAFT_64792* (OW) tobacco seedlings exhibited higher photosynthetic rates and plant growth in alleviating BDE-3 stress than the wild type (WT). Vacuoles and the cytoplasm are the primary transportation and distribution storage organs for BDE-3. The deposition of BDE-3 in leaf cytoplasm and vacuoles prevented it from reentering the surrounding medium. The findings have substantial implications for using phyllosphere microbiome to improve plant stress tolerance.

## 1 Introduction

Air pollution is an important global concern, with atmospheric pollutants causing harmful effects on human health. Polybrominated diphenyl ethers (PBDEs) as persistent organic pollutants (POPs) have attracted much attention in recent years due to their persistence, toxicity, and probable bioaccumulation (Jiang et al., 2019). The halogenated flame retardants of PBDEs are widely utilized in daily living, thus being widely distributed in the environment and causing adverse health effects (Wu et al., 2023). Previous research reveals debromination of PBDEs by photolysis and bioremediation in the natural environment (Yokota et al., 2022). Plant-associated bacteria have been widely used to remove polycyclic aromatic hydrocarbon (PAH) (Gréau et al., 2023). These microbes, especially endophytic phyllosphere, significantly contribute to the adsorption and removal of POPs (Jangra et al., 2024; Tan et al., 2022; Wróblewska and Jeong, 2021). The metabolites of halogenated or aromatic compounds are directly degraded by microbiota intracellular enzyme activity, such as glutathione-S-transferase and hydrolase (Zhang et al., 2022). The microbiota plays a fundamental role in carbon source conversion, nitrogen fixation and nitrification, and regulating bioremediation pollutants (Laird et al., 2020). Phyllotropic yeast strains of *W. anomalus* on *P. tomentosa* have been successfully used as bioremediation tools and to investigate the bioremediation of PBDEs in recent studies (Cai et al., 2022). According to research, the primary biodegradation pathway of PBDEs includes debromination, hydroxylation, and cleavage of the diphenyl ether bond (Liu et al., 2017). The degradation of PBDEs, including the genes and enzymes, is responsible for biodegradation, and the molecular mechanism of degradation remains unsolved.

Xenobiotics detoxification is a complex process involving transformation and compartmentation (Coleman et al., 1997). ABC (ATP-binding cassette) proteins are a large and ubiquitous family, most of which mediate transport across biological membranes to sequester into the vacuole (Lefèvre et al., 2015). Several previous studies found that ATP binding cassette (ABC) transporters proteins may also critical to the distribution of a wide range of exogenous compounds. Compartment membranes or transport vesicles (cytoplasmic and Golgi vesicles) have been reported to store a variety of soluble and insoluble phenolic compounds (Shahidi and Yeo, 2016). ABC transporters improved yeast resistance to toxic compounds, such as fungicides and heavy metal (Karamanou and Aliferis, 2020; Nagy et al., 2006). To date, the study of POP accumulation and detoxification mechanisms has been overlooked.

Yeasts have been shown to remove toxic compounds and other metals from the environment (Wysocki and Tamás, 2010). The yeast was found to have a higher pollutants removal efficiency (Aibeche et al., 2022). The yeast of phyllospheric *Wickerhamomyces anomalus* was isolated from the leaves of *Populus tomentosa*. However, evidence to support the use of phyllospheric yeast in the bioremediation of organic pollutants is limited. In this study, 4-monobrominated diphenyl ether (BDE-3) is used as a model for studies on the molecular mechanisms of PBDE uptake, toxicity, and detoxification. RNA sequencing (RNA-seq) was used to identify BDE-3-responsive differentially expressed genes and the essential detoxification processes involved in phyllospheric yeast. We selected the high expression gene of the ABC transporter to unveil the molecular mechanisms of detoxification and tolerance of phyllospheric yeast response to BDE-3 stress. These results provided insight into the mechanisms underlying other PBDE congeners’ detoxification.

## 2 Materials and methods

### 2.1 Chemicals and materials

BDE-3 was purchased from Solarbio (Beijing, China). Total RNA kit (Tiangen Biotech Co., Ltd., Beijing, China) and quantitative polymerase chain reaction (qPCR) chemistries (Tiangen Biotech Co., Ltd., Beijing, China) were used for the detection and quantitation of differential gene expression. All other reagents were obtained from Sigma Aldrich (St. Louis, MO, USA). Tobacco tissue culture seedlings were cultured as previously described (Cai et al., 2015).

### 2.2 Yeast strain and culture conditions

*Wickerhamomyces anomalus*, a yeast strain, was isolated from *P. tomentosa* leaves and performed well in BDE-3 stress. Yeast cells were preserved in glycerol at −80°C. To generate a fresh yeast inoculum, one loop of yeast colony was transferred into 10 mL of LB medium in a 50-mL flask and incubated aerobically overnight in a shaker at 28°C and 200 rpm for 12 h, until it reached the logarithmic growth stage. The yeast strain was collected in a centrifuge solution (5,000 rpm, 5 min, 4°C) and rinsed with LB medium three times. The residual nutrient medium was removed using the centrifuge solution (5,000 rpm, 5 min, 4°C). The acquired yeast strain was cultivated in LB medium, and the absorbance (OD_600_) value of yeast liquid was adjusted to 0.5.

### 2.3 BDE-3 treatment

In the treatment, the final concentration of BDE-3 in LB medium was 3 mg/L. The same volume of yeast inoculum was inoculated into 50 mL of LB medium supplemented with and without BDE-3 in 250-mL flasks. Then, yeast was incubated with shaking at 180 rpm and 30°C for 24 h. The yeast cells were washed three times with sterile water, frozen in liquid nitrogen, and kept at −80°C.

### 2.4 RNA extraction and sequencing

Yeast cells were ground in liquid nitrogen. Total RNA was extracted from the samples using the RNA simple Total RNA kit (Tiangen Biotech Co., Ltd., Beijing, China) according to the manufacturer’s instructions. The acquired RNA products were measured and validated using NanoDrop (Thermo Fisher Scientific Inc.) and 1% agarose gel electrophoresis prior to next-generation sequencing library creation. Library sequencing was performed using the Illumina Novaseq 6000 sequencing platform.

#### 2.4.1 Differential gene expression analysis and enrichment analysis

Nine candidates of ABC genes were chosen to validate the transcriptome data using qPCR. The list of genes and primers is presented in **Supplementary Table S1**. The Thermal Cycler Dice Real-Time System was used for both amplification and detection. The reaction mixtures consisted of 8 μL of SYBR Premix Ex Taq II (Tiangen Biotech Co., Ltd., Beijing, China), 0.5 μL of each primer (10 μM), and 1 μL of synthesized cDNA, totaling 10 μL. Following the initial denaturation at 95°C for 10 s, the PCR procedure consisted of 40 cycles of 95°C for 10 s, 55°C for 10 s, and 72°C for 10 s. The actin gene was used as an internal gene expression control for the sequence described by Li et al. (2020). The entire experiment was repeated three times. The 2^−ΔΔ^CT value was generated and utilized to indicate the relative expression level of target genes. Gene Ontology (GO) terms annotating enriched significant genes with *p*<0.05. Kyoto Encyclopedia of Genes and Genomes (KEGG) annotation was used to discover the major metabolic pathways of extract genes with substantial differential expression.

### 2.5 Construction of *WICANDRAFT_64792* transgenic tobacco to subcellular localization assay

The full-length coding sequence (CDS) in *WICANDRAFT_64792* was amplified by PCR from complementary DNA from the corresponding wild-type (WT) tobacco. Target fragment was cloned into pCAMBIA1302-GFP vector with little modification. The fragment containing the 35S promoter, the CDS of *WICANDRAFT_64792*, was cloned into the pCAMBIA1302-GFP vector to generate the overexpression vectors, resulting in pCAMBIA1302-64792-GFP. After confirmation of the target sequence, the construct vectors were transformed into WT tobacco seedlings using *Agrobacterium*-mediated transformation. The protoplast from WT and overexpressing *WICANDRAFT_64792* (OW) tobacco seedlings was obtained following the method described by the plant protoplast preparation and transformation kit (RTU4052) (Real-Times Beijing Biotechnology Co., Ltd., Beijing, China). The OW tobacco leaves and protoplast were used in the subcellular localization assay. The subcellular localization of WICANDRAFT_64792 protein was investigated using a confocal laser microscope (LSM 900, Zeiss).

### 2.6 Quantitative and qualitative analysis of BDE-3

BDE-3 concentrations and metabolites in yeast and various tissues and organs from WT and OW tobacco seedlings with BDE-3 stress were measured using GC-MS (Agilent 6890 inert MSD), as described previously (Peng et al., 2013). The sap from xylem and phloem was collected using the method described in the study by Zhang et al. (2024). Apoplasts were prepared as previously described by Gentzel et al. (2019). Shimaoka et al. (2004) reported the isolation of vacuoles from suspension-cultured cells. All the tissues and organ samples collected from WT and OW tobacco seedlings (*N* = 9 individuals per species) were combined as one sample with three replicates.

The samples were freeze-dried and crushed. Extraction and purification were treated with n-hexane and dichloromethane simultaneously. The carrier gas is high-quality helium, with a 30-m HP-5 quartz capillary column. The injection volume was 1 μL, and the inlet temperature was 250°C. The temperature gradient started at 80°C and maintained for 1 min, then increased at 20°C/min to 280°C for 5 min. The shunt ratio is set to 25:1, with a flow rate of 20 mL/min. Each component’s peak area was quantitatively analyzed utilizing the internal standard technique.

### 2.7 Statistical analysis

Statistical analyses were performed using the SPSS software SPSS 20.0, and curve fitting was done with OriginPro 2024b. All data were checked for normality before analyses of variance (ANOVAs). Differences between means were analyzed by Duncan’s tests following two-way ANOVAs to evaluate BDE-3 and heavy metal stress effects. All statistical tests were considered significant at *p* < 0.05 throughout the investigation.

## 3 Results and discussion

### 3.1 Growth inhibition and metabolite identification of yeasts under BDE-3 treatment

**Figure 1:**
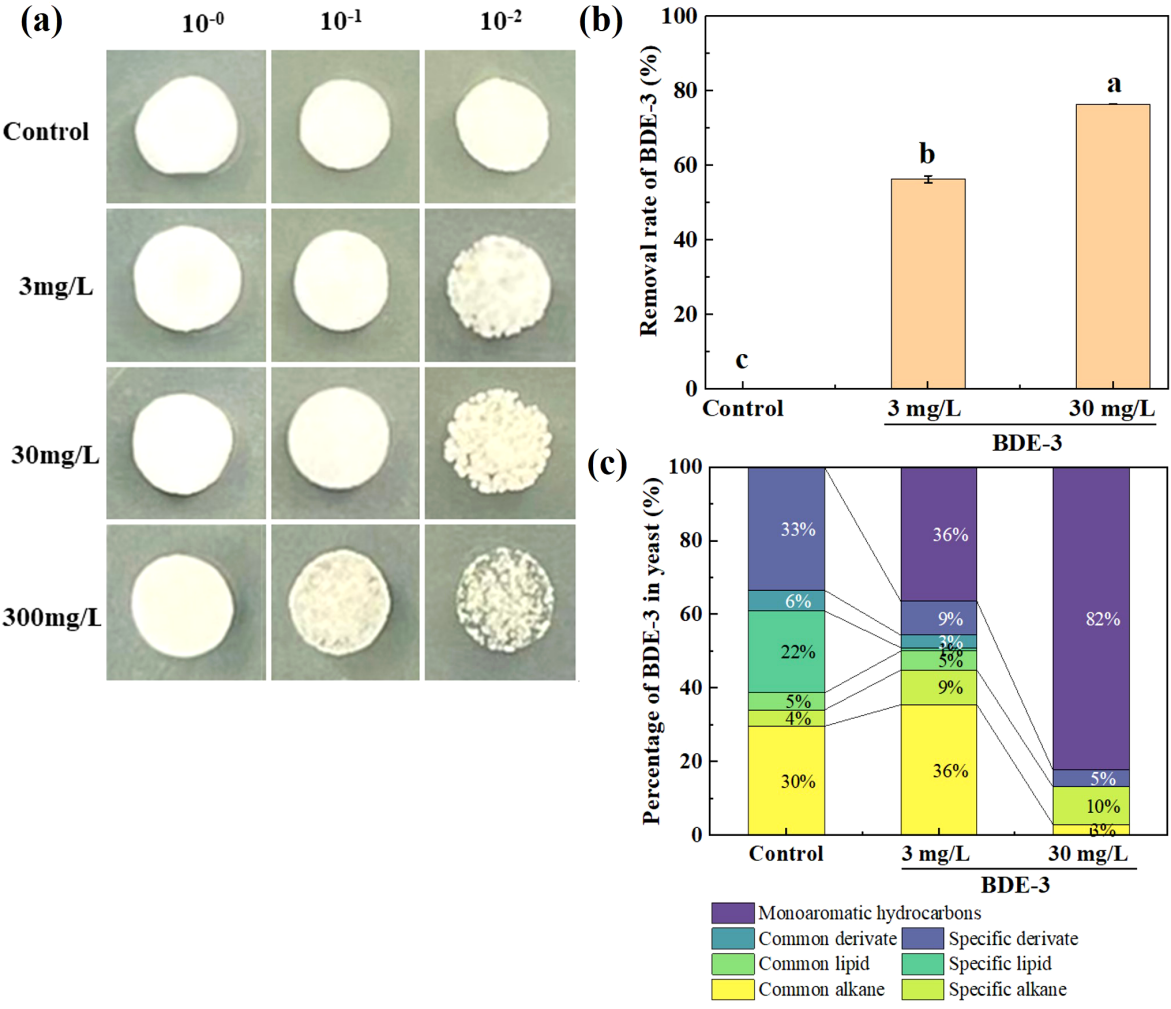
The yeast cells treated with 0, 3, 30, and 300 mg/L BDE-3. **(a)** The yeast spot assay with BDE-3 treatment. **(b)** Removal rate of BDE-3 in yeast cells. **(c)** The percentage of main components in yeast cells. Different letters meant significant differences in different treatments (*p* < 0.05).

The yeast cells were cultured with 0, 3, 30, and 300 mg/L BDE-3 in LB medium at 28°C for 48 h. As demonstrated in **Figure 1a**, we conclude that there was no significant effect on growth of yeast with environmentally relevant concentrations of BDE-3 (3 mg/L) stress (*p* < 0.05). Yeast cell proliferation was significantly repressed by a high dose of BDE-3 (30 and 300 mg/L) stress (*p* < 0.05). A low dose (3 mg/L) and a high dose (30 mg/L) of BDE-3 were selected for the analysis of removal efficiency. Yeast had high removal efficiency of BDE-3, with removal efficiencies of 56% and 71% at different concentrations (3 mg/L and 30 mg/L), respectively (**Figure 1b**). A high dose of BDE-3 (30 mg/L) exhibited higher removal efficiencies and identified key genes associated with bioremediation BDE-3 (30 mg/L) through transcriptome analysis with BDE-3 stress.

Significant changes in cell composition could potentially influence unique metabolic patterns with or without 3 and 30 mg/L BDE-3 stress (**Figure 1c**). The abundance of metabolites depicted that some metabolites varied significantly between BDE-3 (3 mg/L) stress and LB medium. A total of 40 metabolites were detected in LB medium. The metabolites were negatively correlated with total BDE-3 content. In yeast cells, 3 mg/L BDE-3 stress caused changes in the quantity of 45 metabolites, whereas 30 mg/L BDE-3 stress identified 11 metabolites. These metabolites were further classified into common alkane, specific alkane, common lipid, specific lipid, common derivate, specific derivate, and monoaromatic hydrocarbons. The metabolites in BDE-3 treatment were significantly enriched in monoaromatic hydrocarbons in yeast cells, including 1,2-bis(trimethylsilyl)benzene, phenol, butylphenol, benzene, and benzoquinone. With the increase in BDE-3 concentration, the metabolites of monoaromatic hydrocarbons and specific alkane have significantly increased. Furthermore, the metabolites promoted particular alkane responses, such as tetracontane, 1-bromodocosane, tetracosane, dotriacontane, tetratetracontane, nonadecane, octadecane, and eicosane. However, the specific lipid metabolites were significant reduced in yeast cells with BDE-3 stress, such as 6-tetradecanesulfonic acid, cis-9-hexadecenoic acid, oleic acid, n-hexadecanoic acid, and hexadecanoic acid. The abundance of metabolites such as heptadecyl trifluoroacetate, 13-docosenamide, benzeneacetyl chloride, sulfurous acid, phthalic acid, and dibutyl phthalate was positively correlated with the reduction in the medium’s total BDE-3 content. The metabolites affected yeast cell growth, which showed obvious regulation in the degradation of BDE-3.

### 3.2 Transcriptome analysis of yeast under BDE-3 treatment

The complete results of the sequencing of the transcripts of six samples (three with and three without BDE-3 treatment) are provided in **Supplementary Table S2**. A total of 25.74 Gb of clean data were collected. The clean data in all samples exceeded 6.08 Gb. The effective data amount of each sample ranged from 4.11 to 4.42 Gb, the Q30 base distribution varied from 94.37% to 94.69%, and the average GC content was 47.74%. The reads were successfully matched to the reference genome, with an alignment rate of 80.05% to 83.5%. Sequences with numerous alignment places on the reference genome accounted for 1.21% to 3.03%, whereas those with unique alignment positions accounted for 77.14% to 82.23%. The analysis identified 3,063 differentially expressed genes between with and without BDE-3 stress. Further analysis identified 1,611 upregulated and 1,452 downregulated genes in BDE-3 stress compared to control (**Supplementary Figure S1a**).

GO enrichment was used to analyze the biological function of bioremediation BDE-3. A total of 1,608 differentially expressed genes were annotated into GO categories for further analysis (**Supplementary Figure S1b**). The expression of genes encoding biological process and cell component was significantly enriched. Biological process was shown to regulate reproductive process, response to stimulus, component organization or biogenesis, localization, metabolic process, and cellular process. Cell component was associated with the membrane-enclosed lumen, protein-containing complex, organelle, membrane, and cell parts and promoted the detoxification process of BDE-3 stress. The molecular function was associated with the structural molecule activity, molecular function regulator, transcription regulator activity, transporter activity, binding, and catalytic activity. In this study, we found that many differentially expressed genes regulate cell composition, including genes that encode biological regulation (16.04%), metabolic process (37.31%), multi-organism process (0.62%), reproductive process (2.8%), cellular component (13.74%), cellular process (46.33%), and developmental process (1.12%) to improve BDE-3 tolerance. The data were analyzed using metabolite set enrichment analysis and pathway analysis using the KEGG library. The results showed that at least five pathways were involved in amino acid metabolism: degradation, replication and repair, cell growth/death, transport, and catabolism (**Supplementary Figure S1c**). The transport and catabolism pathways were associated with the detoxification process of BDE-3.

### 3.3 Differentially expressed gene analysis for BDE-3 detoxification

Among 697 genes screened, 245 genes were upregulated and 154 were downregulated by GO and KEGG annotation analysis with BDE-3 stress. These genes were highly enriched in cell-wall binding, complexation, vacuolar sequestration, and efflux.

#### 3.3.1 Cell-wall binding process

**Figure 2:**
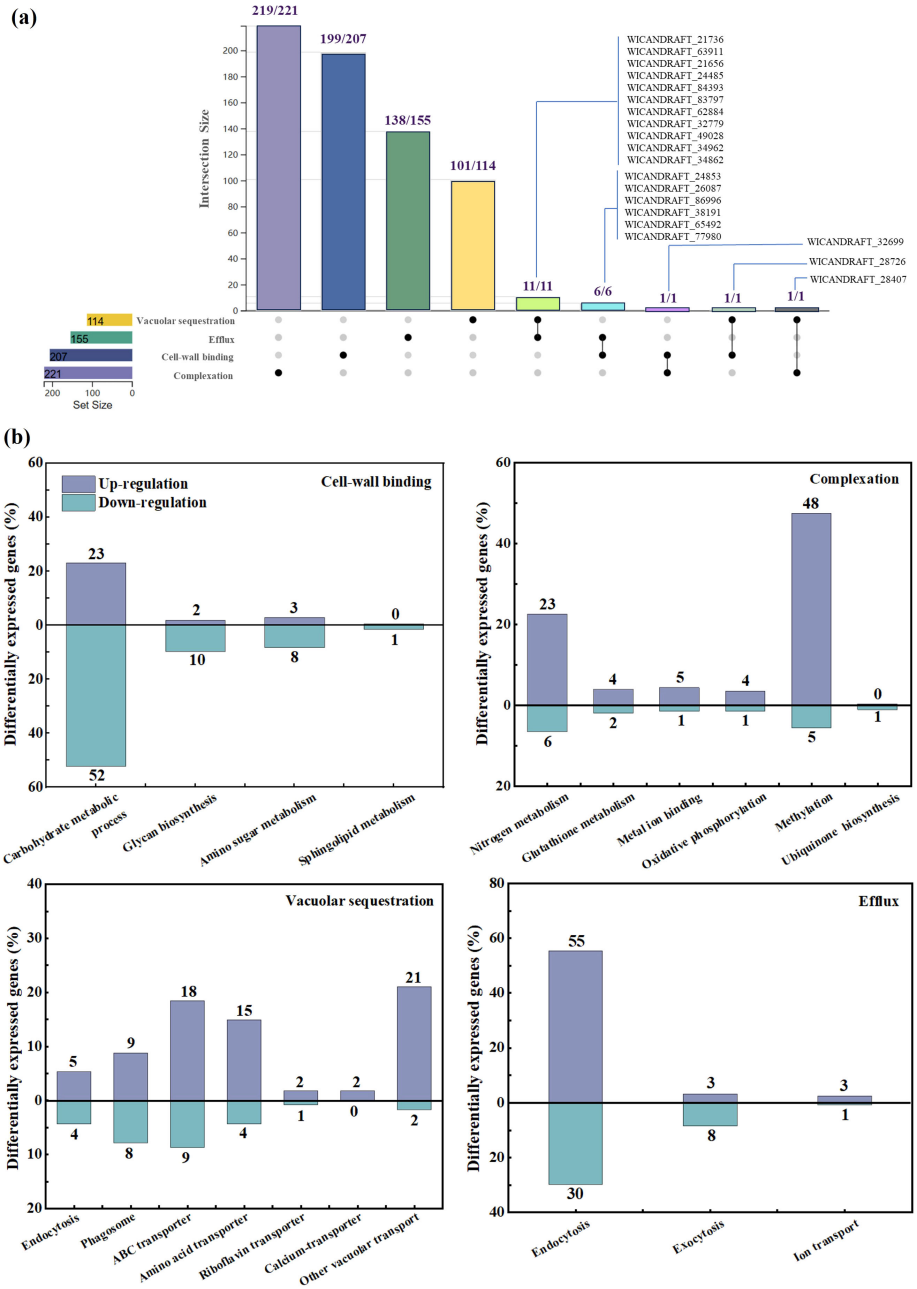
Differentially expressed genes in transcriptome and analysis. **(a)** Venn diagram of the number of differentially expressed genes with and without BDE-3. **(b)** Transcriptomic investigation of the detoxification process during BDE-3 treatment, including cell‐wall binding, complexation, vacuolar sequestration, and efflux.

**Figure 3:**
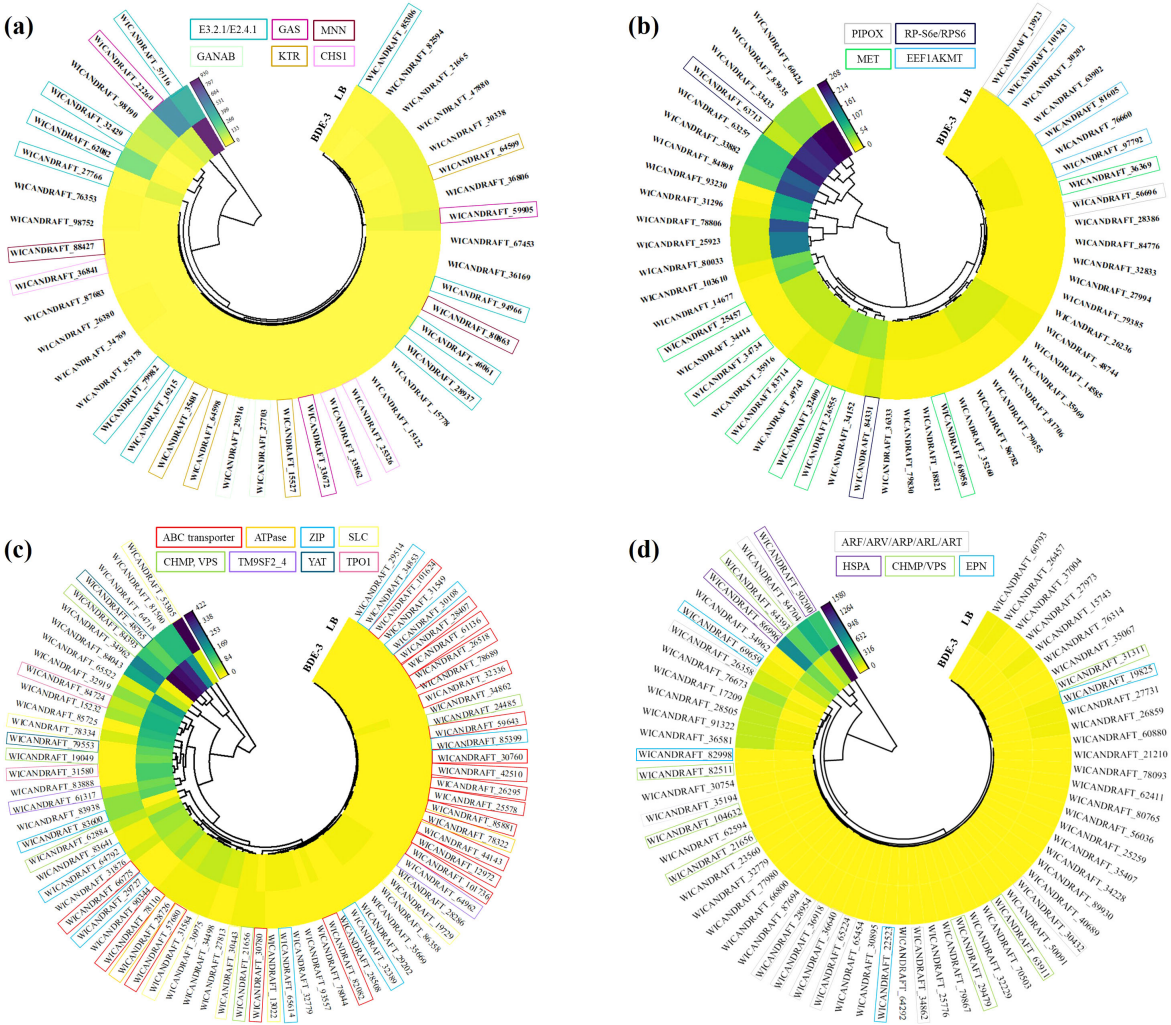
Identifying differentially expressed genes throughout the detoxification process. **(a)** Cell‐wall binding, **(b)** complexation, **(c)** vacuolar sequestration, and **(d)** efflux.

In the cell‐wall binding process, more than 28 genes were upregulated and more than 71 genes were downregulated (**Figure 2a**). The surface of the yeast cell wall contained a range of functional groups (such as carboxyl, hydroxyl, aldehyde, or amino groups) that can act as binding sites for xenobiotics, decreasing their uptake. Exposure to BDE-3 affects cell-wall binding in glucose metabolism, glycan biosynthesis, amino sugar metabolism, and sphingolipid metabolism, as well as related genes *E3.2.1/E2.4.1*, *GAS*, *MNN*, *GANAB*, *KTR* and *CHS1* (**Figure 3a**). The functions of these genes focused on factors that regulate amino sugar and nucleotide sugar metabolism, hydrolase, transferase, N-glycan biosynthesis, N-glycan biosynthesis, and O-glycan biosynthesis (De Coninck et al., 2024).

#### 3.3.2 Complexation process

In the complexation process, a total of 100 significantly expressed genes were identified with 84 upregulated and 16 downregulated genes (**Figure 2b**). Intracellular detoxification was a highly complicated process that involves both xenobiotic transport and detoxification. Xenobiotics enter the cytoplasm, undergo hydrolysis or oxidation, and then conjugate to low-molecular-weight ligands, peptides, or proteins. The complexation may occur through nitrogen metabolism, glutathione metabolism, metal ion binding, oxidative phosphorylation, methylation and ubiquinone biosynthesis with BDE-3 stress, relevant genes such as *PIPOX, RP-S6e/RPS6, MET* and *EEF1AKMT* (**Figure 3b**). BDE-3 that enters plants synthesizes chelating peptides and complexes, which reduces BDE-3 reactivity and toxicity in plants.

#### 3.3.3 Vacuolar sequestration process

The vacuolar sequestration profiling identified a total of 100 differentially expressed genes (72 upregulated and 28 downregulated) (**Figure 2c**). Following xenobiotic complexation, conjugates deposited in the vacuole can be further metabolized (Coleman et al., 1997). Complexation implicated in integral components of membrane, xenobiotic transport, and ATP binding-related genes such as ABC transporter, *ATPase*, *ZIP*, *SLC*, *CHMP*, *VPS*, *TM9SF2_4*, *YAT*, and *TPO1* was induced by BDE-3 (**Figure 3c**). A wide range of substrates are pumped actively by ABC transporters across cellular membranes (Amawi et al., 2019). The drug transporter proteins of the ABC transporter were critical to the distribution of endogenous compounds and xenobiotics. The ABC transporter is associated with the regulation uptake and transport of BDE-3, including *WICANDRAFT_28407*, *WICANDRAFT_25578*, *WICANDRAFT_64792*, *WICANDRAFT_42510*, *WICANDRAFT_101624*, *WICANDRAFT_66775*, *WICANDRAFT_28028*, *WICANDRAFT_44143*, and *WICANDRAFT_85881* (Panda et al., 2025). The ABC transporter represents a major pathway for the vacuolar sequestration of hydrophilic GSH-conjugated BDE-3, xenobiotics, and endogenously produced toxins for degradation into vacuoles (Wysocki and Tamás, 2010). However, how this protein contributes to BDE-3 tolerance remains to be established.

#### 3.3.4 Efflux process

Complexation of xenobiotics from inside organelles pump out low toxic compounds from yeast cells to detoxify (Teelucksingh et al., 2020). In the efflux process, 61 upregulated genes and 39 downregulated genes were identified after BDE-3 stress (**Figure 2d**). This process elevated the expression of endocytosis, exocytosis, and ion transport genes such as ARF/ARV/ARP/ARL/ART, HSPA, CHMP/VPS, and EPN proteins (**Figure 3d**). The ADP-ribosylation factor (ARF) superfamily of regulatory GTPases (*ARF*, *ARV*, *ARP*, *ARL*, and *ART*) regulated a wide range of cellular functions, including vesicular traffic and lipid metabolism in cell biology (Drost et al., 2015).

**Figure 4:**
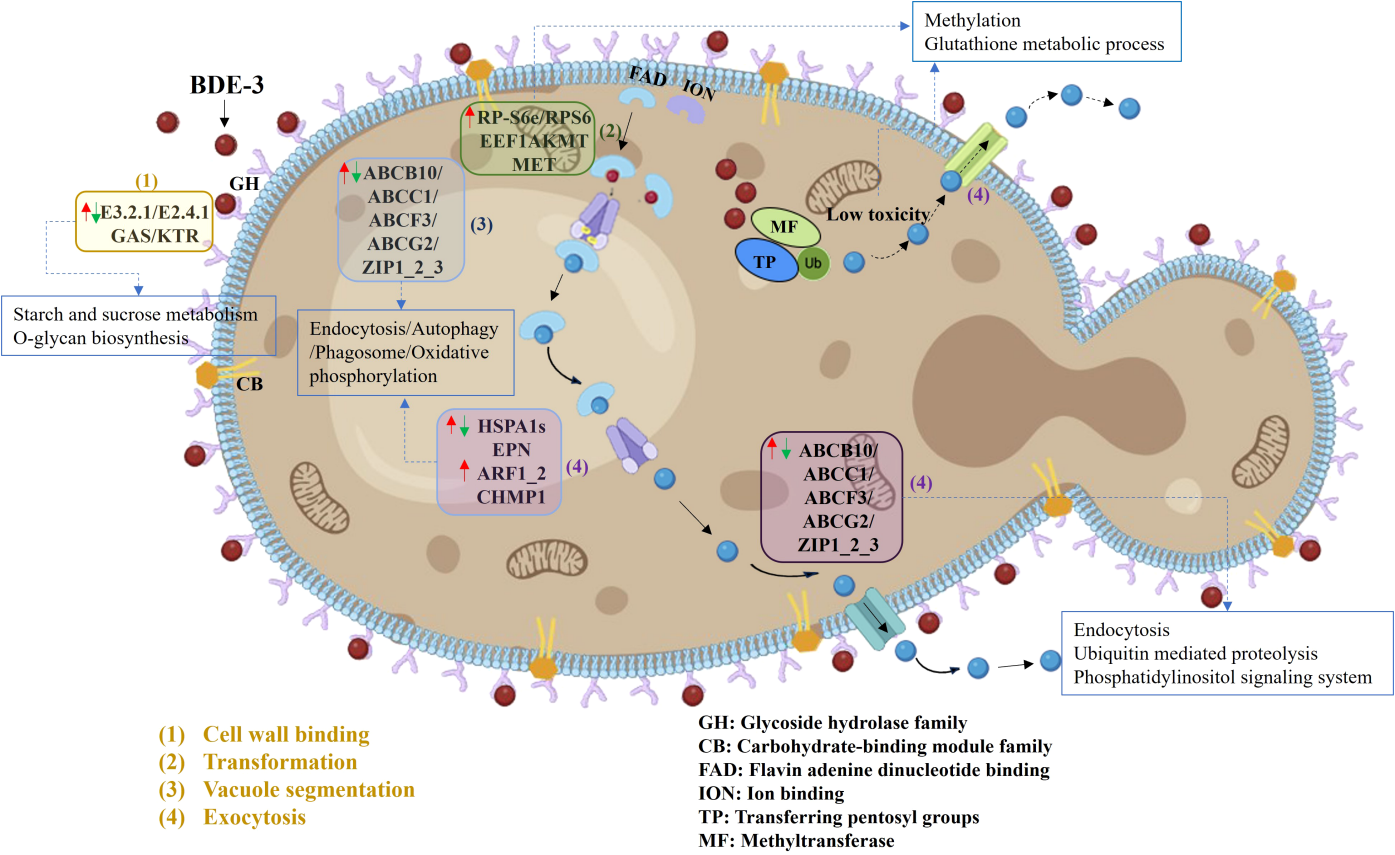
Detoxification of BDE-3 in yeast cells via ABC transporter genes (|log2FC| >1, *p* < 0.05). GH refers to glycoside hydrolase family; CB refers to carbohydrate-binding module family; FAD refers to flavin adenine dinucleotide binding; ION refers to ion binding; TP refers to transferring pentosyl groups; MF refers to methyltransferase. The plasma membrane had seven ABC transporter genes, including the plasma and the vacuolar membranes. The upwards arrow (red arrows) indicated increase in gene expression. The descending arrow (green arrows) indicated decrease in gene expression.

The differentially expressed genes were the first to describe BDE-3 detoxification processes of cell-wall binding, complexation, vacuolar sequestration, and efflux (**Figure 4**). Cell-wall adsorption genes were identified by KEGG analysis, genes related to cell-wall binding for reducing xenobiotic toxicity. A series of genes (*E3.2.1/E2.4.1*, *GAS*, and *KTR*) were related to enzyme activity and gene expression from starch and sucrose metabolism, as well as O-glycan biosynthesis. These genes play a physiological role in cell-wall metabolism and the activation of bioactive substances (Horikoshi et al., 2022). Under BDE-3 stress, differentially expressed genes were associated with chelation, including flavin adenine dinucleotide binding, ion binding, pentosyl group transfer, and methyltransferase. Upregulation of *RP-S6e/RPS6*, *EEF1AKMT*, and *MET* genes encoding activities was associated with methylation and GSH metabolism, as well as cell damage reduction. Genes encoding functions relevant to BDE-3 transport were identified. The expression analysis identified a series of genes (*ABCB10*, *ABCC1*, *ABCF3*, *ABCG2*, and *ZIP*) that were differentially expressed. These genes were related to endocytosis, autophagy, phagosomes, and oxidative phosphorylation with BDE-3 stress. The ABC transporter superfamily associated with drug transport in yeast mainly regulates multidrug resistance. Overexpression efflux pumps of *ABCG/PDR* genes were linked to azole resistance (Kumari et al., 2021). Some genes were activated for endocytosis, ubiquitin-mediated proteolysis, and the phosphatidylinositol signaling pathway. Efflux to BDE-3 was mostly associated with the following genes: *ABCB10*, *ABCC1*, *ABCF3*, *ABCG2*, *ZIP1_2_3*, *HSPA1s*, *EPN*, *ARF1_2*, and *CHMP1*. The function of these genes with BDE-3 stress remains ambiguous in yeast.

### 3.4 Expression pattern of ABC transporter genes

**Figure 5:**
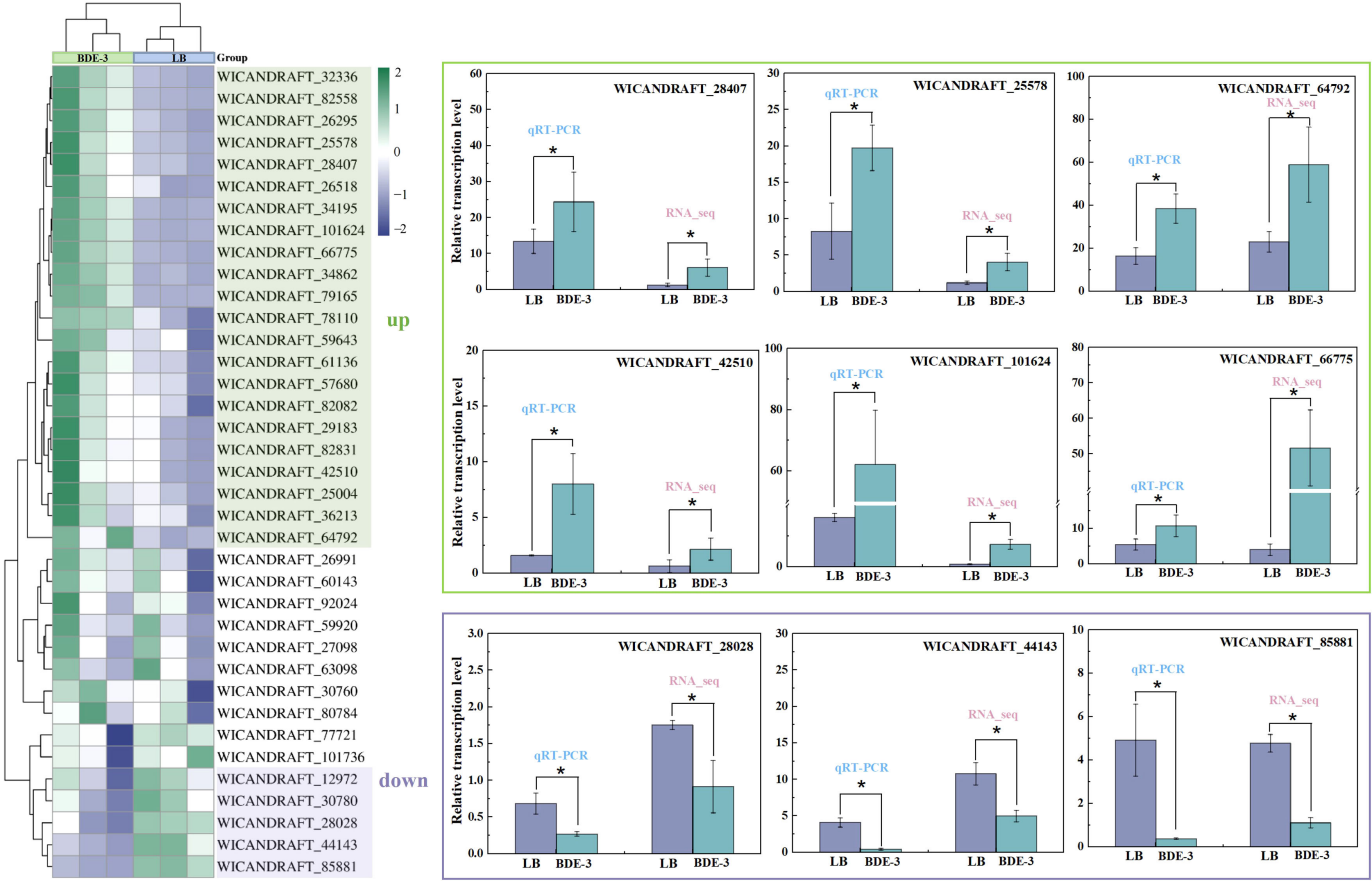
RT-qPCR validations of the results obtained in RNA-seq analysis. The 2^−ΔΔCT^ was performed, and two-way ANOVA with Duncan’s test was utilized for significant analysis of the RT-qPCR at *p < 0.05. The absolute log2 fold change value greater than one was judged statistically significant in the RNA-seq study. Three biological replicates were constructed, with results shown as means ± SD.

The recent investigation had shown that the ABC transporter genes was involved in the detoxification and excretion of heavy metals (Kumari et al., 2021). However, the mechanism of absorption, distribution, metabolism and excretion of BDE-3 remain unclear. In this study, transcriptomic analysis identified 599 differentially expressed genes, of which 37 were ABC transporter-related genes, and participated in BDE-3 detoxification. The expression patterns of the ABC transporter in BDE-3 treatment were determined by qPCR, as shown in **Figure 5**. RT-qPCR was used to verify the correctness of the transcript sequence and expression data. These target genes participated in the transport of BDE-3 into vacuoles at extremely significant expression levels for further research of the specific functions of ABC transporter family genes of the phyllospheric *W. anomalus*. The expression levels of six genes—*WICANDRAFT_28407, WICANDRAFT_25578, WICANDRAFT_64792, WICANDRAFT_42510, WICANDRAFT_10126* and *WICANDRAFT_66775*—were significantly upregulated with BDE-3 stress (*p* < 0.05). Compared with normal growth conditions, three genes were significantly downregulated, associated with the expression levels of *WICANDRAFT_28028*, *WICANDRAFT_44143*, and *WICANDRAFT_85881* (*p* < 0.05). The expression levels of these differentially expressed genes assessed by RT-qPCR were similar to the transcriptome data, demonstrating high reliability to transcriptome results.

### 3.5 Overexpressing target ABC transporter gene increased BDE-3 uptake

**Figure 6:**
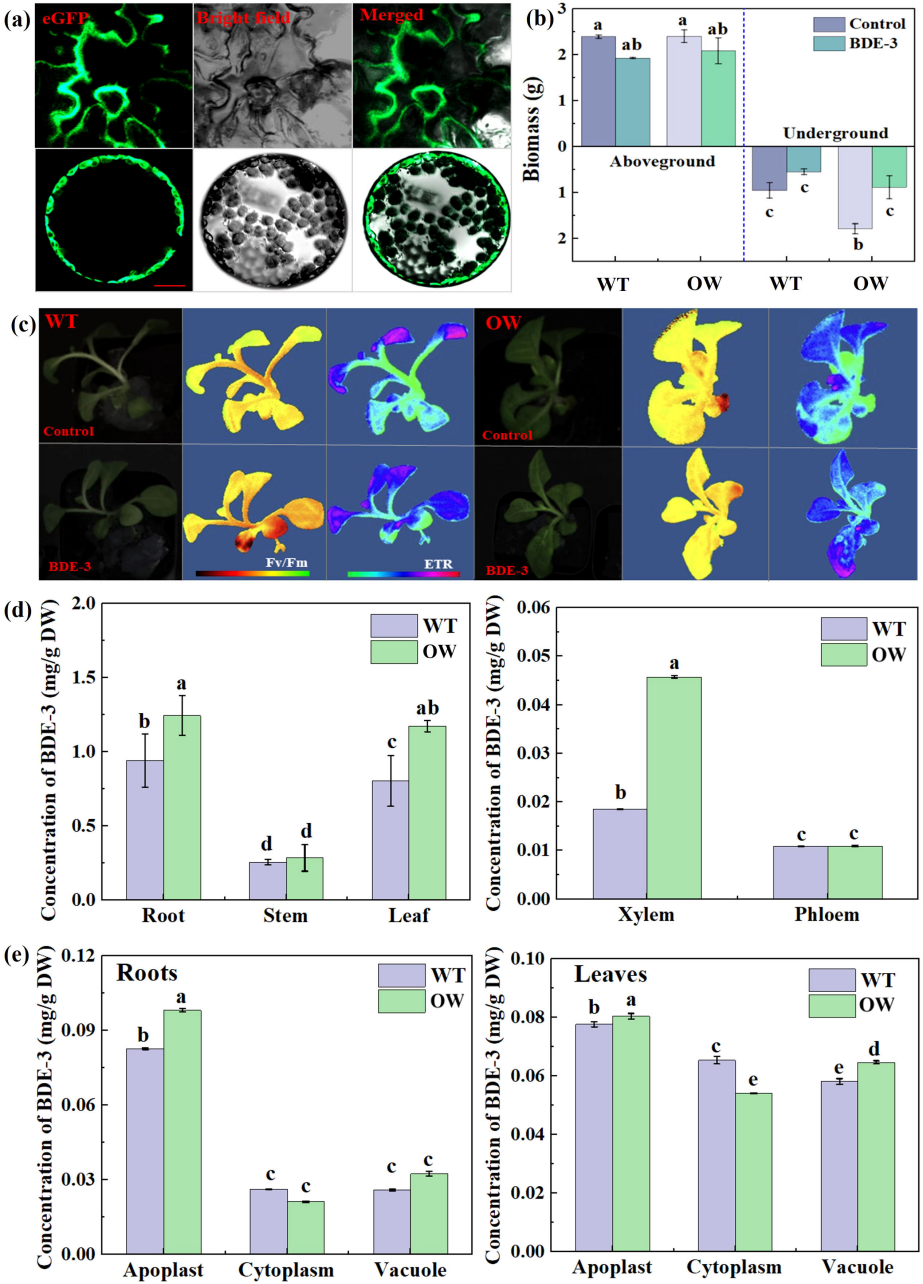
Subcellular localization, biomass, and photosynthetic parameters of overexpressed target gene under BDE-3 stress. **(a)** Subcellular localization of GFP-tagged WICANDRAFT_64792 (64792-GFP) in tobacco leaf protoplasts. The photos were obtained using the GFP channel or combined images from two channels. Scale bar: 100 μm. **(b)** Changes in biomass from wild type (WT) and overexpressing *WICANDRAFT_64792* (OW), including aboveground and belowground biomass. **(c)** Photosynthetic activity of WT and OW, including Fv/Fm and ETR. **(d)** The concentration of BDE-3 in roots, stems, leaves, xylem, and phloem. **(e)** The concentration of BDE-3 in apoplast, cytoplasm, and vacuole. The values represent the means ± SD (*n* = 3). Different letters meant significant differences between WT and OW.

To assess the bioremediation potential of the ABC transporter gene from phyllospheric yeast, the biological impact on plant development and the subcellular location of the target gene of *WICANDRAFT_64792* were analyzed. To determine the subcellular localization of target WICANDRAFT_64792, we transiently generated eGFP fused to the C-terminus of protein (as pCambia-64792-eGFP) in tobacco mesophyll protoplasts driven by the CaMV 35S promoter. The green fluorescence of 64792-eGFP overlapped with the emission from the plasma membrane of tobacco mesophyll cell protoplasts (**Figure 6a**). These findings show that WICANDRAFT_64792 protein was located at the plasma membrane. It appears to be responsible for the transport of BDE-3 out of the cell or segmentation into vacuoles. In the previous research, a subfamily of ABC exporters were involved in pleiotropic drug resistance (PDR) and in the cellular efflux of a wide variety of drugs in the mechanism of defense against toxins (Czarnecka et al., 2022). Thus, whether PDR induced segmentation of BDE-3 into vacuoles was unclear.

The PDR gene of *WICANDRAFT_64792* was overexpressed in tobacco by the *Agrobacterium*-mediated transformation system to determine the molecular functions of ABC transporter genes. The BDE-3 resistance of plants can be evaluated by measuring photosynthesis and biomass. The WT and OW tobacco seedlings were healthy and no significant growth difference was found. The aboveground and belowground biomass revealed no significant difference between WT and OW tobacco seedlings (**Figure 6b, p** < 0.05). Under BDE-3 stress, the biomass of OW tobacco seedlings was significantly higher than that of WT, increasing by 8.7% and 29.63%, respectively (**Figure 6b, p** < 0.05). A low dose of BDE-3 markedly promoted growth to alleviate OW compared with WT (*p* < 0.05). The maximum photochemical efficiency of photosystem II (Fv/Fm) and the electron transport rate (NPQ) were effective markers of plant abiotic stress response. Compared to the Fv/Fm value of WT leaves (0.52), Fv/Fm values of OW tobacco leaves (0.65) increased by 11.37%. With BDE-3 stress, rETR values of WT leaves decreased and OW leaves obviously increased by 13.57%. The values of rETR levels between WT and OW leaves were 24.83 and 32.63 μmol electrons m^2^/s. The OW tobacco leaves have shown considerably improved chlorophyll fluorescence characteristics (Fv/Fm and rETR) in BDE-3 stress (**Figure 6c**).

For the candidate gene of *WICANDRAFT_64792* for BDE-3 stress, we measured the accumulation of BDE-3 in WT and OW tobacco seedlings. BDE-3 concentration was examined in all of the tissues and organs, including leaves, stems, roots of seedlings, and apoplast, cytoplasm, and vacuole of leaves. As shown in **Figure 6**, the content of BDE-3 was decreased in the following order: roots > leaves > stems (**Figure 6d**). BDE-3 content in OW tobacco roots, stems, and leaves was significantly higher than that in WT (*p* < 0.05). Compared with WT, the percentage distribution of BDE-3 in xylem was significantly increased in OW tobacco seedlings. BDE-3 concentration had a nonsignificant effect on phloem (**Figure 6e**). Furthermore, xylem content in root cell was higher than phloem content, indicating long-distance transport of BDE-3 in tobacco through the xylem. BDE-3 concentrations in the apoplast and vacuole of leaves were greater than in roots, but lower in the cytoplasm (**Figure 6e** and d, *p* < 0.05). Concentrations of BDE-3 in the apoplast, cytoplasm, and vacuole of leaves generally followed the following order: apoplast > cytoplasm > vacuole. These results indicated that the first uptake of BDE-3 was though root, then BDE-3 was transported to stems through the xylem, and eventually BDE-3 accumulate into leaf organs via apoplastic pathways. The uptake and accumulation of BDE-3 were facilitated by OW leaf organs, especially vacuoles. Consequently, the increased accumulation of BDE-3 in leaves can be attributed to transporter facilitation through the xylem. Increasing BDE-3 retention in leaves and its transfer from roots to leaves is a crucial process in counteracting BDE-3 toxicity.

## 4 Conclusions

BDE-3 as one of the mono-brominated products of PBDE homologs has gained great attention in bioremediation research of PBDEs (Peng et al., 2013). The bioaccumulation of mono-brominated products results in a prolonged half-life (2 to 10 years) in the environment, causing detrimental effects on natural ecosystems (Li et al., 2008; Paliya et al., 2022). Low brominated diphenyl ethers of BDE-3 are highly bioavailable. As a result, there is an urgent need to analyze the residual dominant homolog of polybrominated diphenyl ethers (BDE-3) in the environment. The behavior of residuals and migrations among organisms has been investigated. In this study, we carried out transcriptomics analysis of yeast with a low dose of BDE-3 stress. We identified many differentially expressed genes by measuring the expression levels in yeast cells with BDE-3 stress. Through GO and KEGG enrichment analysis, the potential detoxification genes were classified into four clusters, namely, cell-wall binding, complexation, vacuolar sequestration, and efflux. Furthermore, the expression patterns of the ABC transporter in BDE-3 treatment were determined by qPCR. Transgenic tobacco lines overexpressing the ABC transporter gene of *WICANDRAFT_64792* were obtained to further specify the mechanism of *WICANDRAFT_64792* gene-mediated BDE-3 transformation in plant.

The ABC transporter gene of *WICANDRAFT_64792* was responsible for BDE-3 accumulation in phyllosphere yeast. The OW tobacco seedlings enhanced BDE-3 absorption ability compared to WT. The results of the tissue culture experiment indicated that the ABC transporter not only mediates BDE-3 absorption by roots, but may also increase BDE-3 transport via the xylem and phloem in tobacco to play a significant role in BDE-3 transport from roots to leaves via the xylem. BDE-3 treatment resulted in the formation and accumulation of a significant variety of metabolites in OW tobacco leaves, particularly enhancing BDE-3 fixation in leaf cytoplasm and vacuoles. The OW tobacco seedlings promoted the deposition of BDE-3 in leaf cytoplasm and vacuole cells. Genes identified from this study can be potential candidates for PBDE congener detoxification and provide candidate genes for phytoremediation.

## Data Availability

The datasets presented in this study can be found in online repositories. The names of the repository/repositories and accession number(s) can be found in the article/**Supplementary Material**.
