# Supplementary material for: Bioremediation potential of low-brominated polybrominated diphenyl by the phyllospheric Wickerhamomyces anomalus
Source: Front Plant Sci. 2025 Jul 11;16:1606962. doi: 10.3389/fpls.2025.1606962 (PMC12289573; doi:10.3389/fpls.2025.1606962)
Supplement: Supplementary file 1 [file DataSheet1.docx]

**Supporting Information**

**Title:** Bioremediation Potential of Low-brominated Polybrominated Diphenyl by the Phyllospheric *Wickerhamomyces anomalus*

**Authors:** Man Cai^a,b,*^, Zheng Xing ^a^, Xiaolei Yuan^a^, Zhangyu Song ^a^, Xiaojing Wang^a^, Kejiu Du^a,b,*^

*^a^ College of Forestry, Hebei Agricultural University, Baoding 071000, China*

*^b^ Hebei Key Laboratory for Tree Genetic Resources and Forest Protection, Baoding 071000, China*

**Corresponding author:**

Man Cai (🖂),

Hebei Key Laboratory for Tree Genetic Resources and Forest Protection, Hebei Agricultural University, 2596 Lokai South Road, Baoding 071000, China;

Telephone:15028290779;

E-mail: caiman@163.com.

Kejiu Du (🖂)

Hebei Key Laboratory for Tree Genetic Resources and Forest Protection, Hebei Agricultural University, 2596 Lokai South Road, Baoding 071000, China;

Telephone: 13833072220;

E-mail: dukejiu@126.com.

**Table S1.** Primer lists used in RT-qPCR reactions.

| **Gene name** | **Gene description** | | **NCBI reference sequence** | **Forward primer (5′-3′)** | **Reverse primer (5′-3′)** | **Product**  **(bp)** | **Cellular localization** |
| --- | --- | --- | --- | --- | --- | --- | --- |
| WICANDRAFT_28407 | ABCC1 | NW_017567111 | | ATTTCCTTGTCCGGTGGTCA | ACCGTTTGGACCCAAGACAT | 150 | plasma membrane |
| WICANDRAFT_25578 | ABCG2 | NW_017567110 | | TTCGTTCTACAGTCCCAGCG | AGAAAGCACCAGCATCACTCT | 134 | plasma membrane |
| WICANDRAFT_64792 | ABCG2 | NW_017567115 | | ATCGGGTTTCGCCATTCACT | TGGTGGACTGAAACGAACCA | 107 | plasma membrane |
| WICANDRAFT_42510 | ABCB7 | NW_017567112 | | TTGTGGGACCCTCTGGTAGT | GGAACAACACCAATTGCTCTCC | 148 | extracellular/cell wall |
| WICANDRAFT_101624 | ABCC1 | NW_017567114 | | ACTGGCCAATTTGTTGCTGG | GAACCAAAGCTCTAGCCAACG | 136 | plasma membrane |
| WICANDRAFT_66775 | ABCF2 | NW_017567110 | | CTTTCGAATCAAGAGCCGCTG | AGCGACACGCATTTTCCAAC | 107 | nucleus |
| WICANDRAFT_28028 | ABC | NW_017567111 | | CCTGCCTTTGGCCATCCTAT | ACGTAGCAGCGGTTCGTATT | 115 | plasma membrane |
| WICANDRAFT_44143 | ABCC1 | NW_017567113 | | GAAGTCGCCAAGCAGGAGAT | TGAATCCAAGGTGCCGAACA | 191 | plasma membrane |
| WICANDRAFT_85881 | ABC | NW_017567115 | | TTTTAGGGGCTTCAGGGTCTG | CTCGAACTGTCAACCCAGGC | 183 | plasma membrane |
| ACT1 | Actin | ------ | | ACATCGTTATGTCCGGTGGT | CCACCAATCCAGACGGAGTA | 142 | ------ |

**Table S2.** Sequencing quality statistics and reference genome comparisons.

| **Sample** | **Raw Reads** | **Clean Reads** | **Q30(%)** | **GC(%)** | **Total mapped** | **Multiple mapped** | **Uniquely mapped** |
| --- | --- | --- | --- | --- | --- | --- | --- |
| LB-1 | 44980620 | 43332726 | 94.53 | 48.02 | 34687967(80.05%) | 1150979(2.66%) | 33536988(77.39%) |
| LB -2 | 45632154 | 43692832 | 94.49 | 48.08 | 35029301(80.17%) | 1324782(3.03%) | 33704519(77.14%) |
| LB -3 | 45766900 | 44200206 | 94.39 | 48.15 | 35673203(80.71%) | 1281496(2.9%) | 34391707(77.81%) |
| BDE-1 | 44385558 | 42900672 | 94.59 | 47.34 | 35824131(83.5%) | 550604(1.28%) | 35273527(82.22) |
| BDE -2 | 42731986 | 41125844 | 94.37 | 47.51 | 34151006(83.04%) | 583708(1.42%) | 33567298(81.62%) |
| BDE -3 | 43767322 | 42174168 | 94.69 | 47.33 | 35192228(83.44%) | 511988(1.21%) | 34680240(82.23%) |


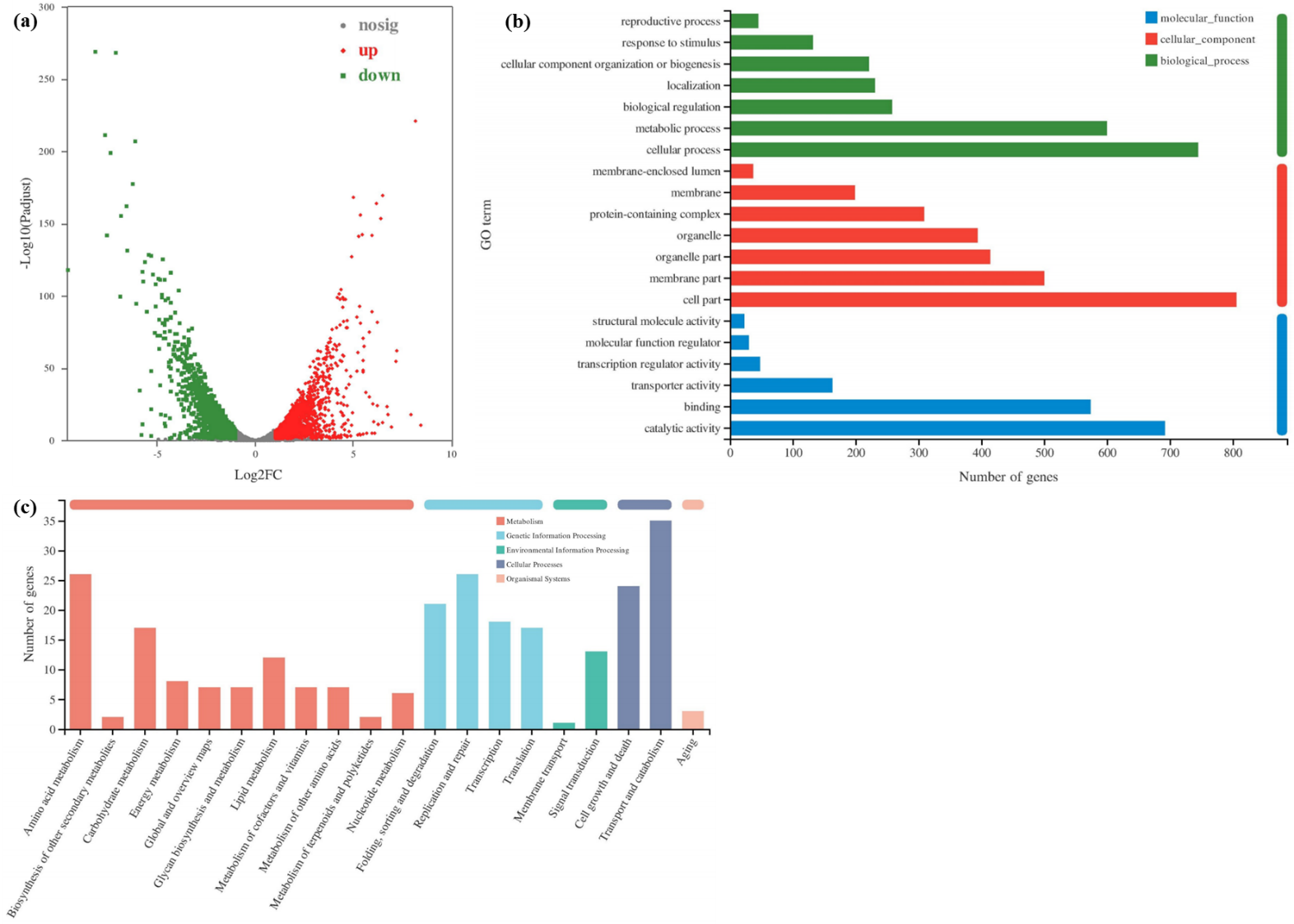


**Fig. S1.** Transcriptional identified differential expression. (a) A cluster diagram of the differentially-expressed lncRNAs. (b) GO enrichment categories. (c) KEGG enrichment categories.

**Acknowledgements**

The data that support the findings of this study have been deposited into CNSA with accession number CNP0007548.
